# In Situ Cross-Linked Porous Starch Microencapsulation Enhances the Colonization of *Lactobacillus* In Vivo

**DOI:** 10.3390/foods14122031

**Published:** 2025-06-09

**Authors:** Xiaojun Zhang, Ying Liang, Hao Bai, Quanhua Huang, Dongming Liu, Guanglei Ma, Xiangrui Liu

**Affiliations:** 1 Department of Pharmacology, Department of Gastroenterology, Second Affiliated Hospital, Zhejiang University School of Medicine, Hangzhou 310058, China; xiaojunzhang92@163.com (X.Z.);; 2 Innovation Center of Yangtze Delta, Zhejiang University, Jiaxing 314100, Chinaguangleima@zju.edu.cn (G.M.); 3 School of Pharmacy, Zhejiang University, Hangzhou 310058, China; 4 Center for Medical Research and Innovation in Digestive System Tumors, Ministry of Education, Hangzhou 310009, China

**Keywords:** delivery system, survival rate, oral gavage, simulated digestion, encapsulation efficiency

## Abstract

In this study, we developed novel porous starch (PS)/*Lactobacillus* (LS) microcapsules via in situ cross-linking with sodium trimetaphosphate (STMP), using *Lactobacillus johnsonii* (LJ), *Lactobacillus acidophilus* (LA), and *Lactobacillus rhamnosus GG* (LGG) as representative strains. Scanning electron microscopy (SEM) revealed that the cross-linked microcapsules (designated as PS/LS-CL: PS/LJ-CL, PS/LA-CL, PS/LGG-CL) formed aggregated structures with denser microarchitecture compared to uncross-linked porous starch/*Lactobacillus* microcapsules (designated as PS/LS: PS/LJ, PS/LA, PS/LGG). The encapsulation efficiencies of PS/LJ-CL, PS/LA-CL, and PS/LGG-CL (79.56%, 78.49%, and 55.96%, respectively) significantly surpassed those of their uncross-linked counterparts (67.92%, 58.68%, and 47.71%, *p* < 0.05). In addition, the cross-linked porous starch microcapsules improved the survival rate of *Lactobacillus* during simulated gastrointestinal digestion and long-time storage. Importantly, the oral gavage of PS/LS-CL, PS/LA-CL, and PS/LGG-CL significantly increased the amount of *Lactobacillus*. The colonization efficiency of all the tested *Lactobacillus* in mice was detected by both gradient dilution plate counting and quantitative real-time PCR (qRT-PCR). These findings indicate the potential function of the in situ cross-linked porous starch microcapsules as a robust delivery system to enhance the colonization of probiotics in vivo.

## 1. Introduction

Emerging evidence from metagenomic studies has established a causal link between gut microbiota perturbations—particularly *Lactobacillus* depletion—and the pathogenesis of inflammatory bowel diseases, metabolic syndrome, and accelerated aging [1,2]. Despite the widespread incorporation of probiotics as functional ingredients in fermented products (global market valuation exceeding USD 87.7 billion) [3], their therapeutic efficacy is fundamentally constrained by gastrointestinal barriers. Specifically, the structural integrity of probiotic cell membranes is compromised upon exposure to gastric acid (pH 1–3) and bile salts (>0.3% *w*/*v*), resulting in a markedly reduced colonization efficiency in the colon [4,5,6]. Engineered encapsulation systems have emerged as a frontier strategy to overcome this bioavailability bottleneck [7]. Current technologies (e.g., alginate–chitosan coacervation, layer-by-layer assembly, film encapsulation) can achieve pH-responsive protection and colon-targeted release, enhancing fecal *Lactobacillus* recovery [7,8]. However, layer-by-layer encapsulation technology commonly employs relatively expensive materials such as polyelectrolytes (e.g., chitosan–alginate systems), proteins (e.g., gelatin, albumin), and polysaccharides (e.g., cellulose derivatives, cyclodextrins), and film encapsulation necessitates the stringent regulation of film thickness, homogeneity, and material properties, elevating production costs due to high-precision deposition equipment and defect mitigation protocols. Furthermore, scalability and cost-effectiveness remain critical limitations for food industrial applications [7,8].

Native starch is a safe and low-cost biopolymer which has been used as a sustainable encapsulation matrix in the food industry [9,10]. Some studies have found that the *Lactobacillus* can effectively adhere to the surface of starch among numerous microbial genera [11,12]. The rough surface of starch can provide attachment sites for probiotics and its glycosidic backbone enables probiotic adhesion via surface glycosidic bond interactions [11,13,14]. Meanwhile, *Lactobacillus* can attach to the surface of starch granules via specific surface proteins, such as glycolytic enzymes and glycoside hydrolases [11]. However, the weak intermolecular forces (e.g., hydrogen bonds and van der Waals interactions) between starch and probiotics result in unstable binding [15], leading to structural disintegration during gastrointestinal transit. Moreover, natural starch granules exhibit limited acid resistance in simulated gastric fluid, and the absence of structural encapsulation results in uncoated probiotics being highly vulnerable to gastric acid and intestinal enzymes.

Porous starch (PS) is a food-grade modified starch with a honeycomb-like structure and tunable pore sizes ranging from 10 nm to 10 μm, which significantly enhances native starch’s adsorption and adhesion properties [16]. PS has been widely utilized as an encapsulating material for various bioactive compounds, including curcumin [17], allicin [18], and essential oils [19]. Recently, Xing et al. and Liu et al. found that the encapsulation rate of PS for *Lactobacillus acidophilus* and *Lactobacillus plantarum* was higher than 60% [20,21]. However, the survival rate of probiotics in the porous starch/probiotic system is comparable to that in the native starch/probiotic system under simulated gastric acid, bile, and heat treatment conditions [22].

The cross-linking modification of starch establishes ester linkages between adjacent starch granules through chemical bonding, thereby promoting intergranular aggregation [23]. This structural reorganization significantly enhances the mechanical integrity and thermal stability of the starch-based matrix [23]. The structural modification hinders acid molecules’ access to hydrolysis sites on starch molecular chains, thereby mitigating the rate of acid hydrolysis [24]. Extensive research has confirmed that cross-linked starch, classified as food-grade RS4-type resistant starch, exhibits gastric acid resistance and small intestinal persistence, and can serve as a prebiotic to promote the colonization of probiotics [25,26]. The primary cross-linking agents employed in starch modification comprise phosphoryl chloride (POCl_3_), sodium trimetaphosphate (STMP), epichlorohydrin, citric acid, and adipic acid [27]. Among these, STMP exhibits distinct advantages over alternative agents such as citric acid and adipic acid, particularly in terms of its mild reaction requirements and the absence of toxic by-products during the cross-linking process [27,28,29].

In this study, we developed a new strategy employing porous starch probiotic encapsulation and in situ cross-linking technique to achieve the effective colonization of *Lactobacillus* in vivo. Three representative *Lactobacillus* species-*Lactobacillus johnsonii* (LJ), *Lactobacillus acidophilus* (LA), and *Lactobacillus rhamnosus GG* (LGG) were used as model strains to develop porous starch/*Lactobacillus* microcapsules (PS/LS-CL) with enhanced stability by implementing a cross-linking strategy. The morphological architecture of the novel microcapsules was characterized using scanning electron microscopy (SEM). In vitro experiments were conducted to investigate the stability of PS/LS-CL under simulated storage conditions and digestive conditions. Additionally, in vivo experiments were performed to further characterize the colonization ability of the new microcapsules.

## 2. Materials and Methods

### 2.1. Materials

Corn starch was purchased from Suzhou Huimai Trading Co., Ltd. (Jiangsu, China). Porous starch was prepared using the enzymatic hydrolysis method previously established by our research group (the enzyme was glycation enzyme, the amount of addition was 800 U/1 g corn starch, the reaction temperature was 50 °C, and the reaction time was 12 h). The porous starch was irradiated under UV conditions for 30 min before encapsulation. *Lactobacillus johnsonii* (LJ), *Lactobacillus acidophilus* (LA, ATCC 13651), and *Lactobacillus rhamnosus GG* (LGG, ATCC 53103) were purchased from Ningbo Testo Biotechnology Co., Ltd. (Zhejiang, China). Sodium trimetaphosphate (STMP), glutaraldehyde, osmium tetroxide, sodium carbonate, pepsin, 0.01 M sodium phosphate buffer (PBS, pH 7.2–7.4), pancreatin, and bile salts were purchased from Shanghai Macklin Biochemical Co., Ltd. (Shanghai, China), and MRS broth and MRS medium were purchased from Qingdao Hi-tech Industrial Park Hope Bio-technology Co., Ltd. (Qingdao, China). RNA isolation Total RNA Extraction Reagent was purchased from Nanjing Novozymes Biotech Co., Ltd. (Nanjing, China), HiScript^®^ II Q RT SuperMix for qPCR (+gDNA wiper) was purchased from Nanjing Novozymes Biotech Co., Ltd. (Nanjing, China), and 2× Universal SYBR Green Fast qPCR Mix was purchased from Ibotek Biotech Co., Ltd. (Wuhan, China).

### 2.2. Preparation of Porous Starch/Lactobacillus Microcapsules

**Figure 1:**
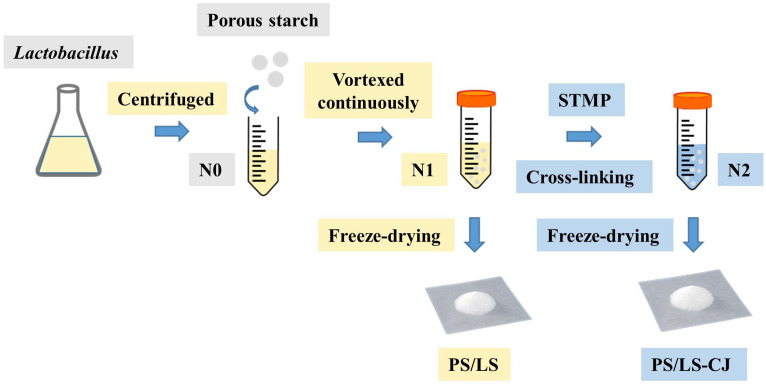
Schematic diagrams for the preparation of PS/LS and PS/LS-CL.

LJ, LA, and LGG bacteria were cultured in MRS broth at 37 °C for 24 h; the precipitates were collected by centrifugation at 6000× *g* for 10 min and washed twice with sterile PBS, and resuspended to obtain LJ, LA, and LGG suspensions with a concentration about 10^9^ CFU/mL. Then, as shown in Figure 1, the bacterial suspension was prepared by combining 10 mL of probiotic culture with 0.1 g of porous starch under sterile conditions. The mixture was subjected to continuous vortex mixing (100 r/min) for 10 h at 4 °C. The supernatant obtained after 10 min of natural sedimentation was subjected to colony plate counting (N1) to calculate the encapsulation efficiency. Following the removal of the supernatant, the mixture was collected by centrifugation at 6000× *g* for 10 min and subsequently freeze-dried at −60 °C for 48 h, yielding the porous starch/*Lactobacillus* encapsulation microcapsules designated as PS/LS: PS/LJ, PS/LA, and PS/LGG.

### 2.3. Preparation of Novel Porous Starch/Lactobacillus Microcapsules

As shown in Figure 1, the porous starch/*Lactobacillus* mixture from Section 2.2 (before natural sedimentation) was centrifuged at 6000× *g* for 10 min. The cross-linking conditions were established based on the method described by Woo et al. [29], with modifications as follows: the resultant pellet was resuspended with 0.15 g sodium trimetaphosphate (STMP) in 10 mL PBS, and the pH of the suspension was adjusted to 9.0 using sodium carbonate, followed by continuous agitation at 45 °C and 50 r/min for 1.5 h. The pH of the suspension was adjusted to 6.8 using acetic acid. After natural sedimentation, the supernatant was collected and plated for colony counting (N2) to calculate the encapsulation efficiency. Following removal of the supernatant, the mixture was collected by centrifugation at 6000× *g* for 10 min and subsequently freeze-dried at −60 °C for 48 h, yielding the novel porous starch/*Lactobacillus* microcapsules designated as PS/LS-CL: PS/LJ-CL, PS/LA-CL, and PS/LGG-CL.

### 2.4. Morphology

Three *Lactobacillus* strains were fixed with 2.5% glutaraldehyde (4 °C, 12 h), post-fixed in 1% osmium tetroxide (1 h), and dehydrated through a graded ethanol series (30–100%, 15 min/step). Three *Lactobacillus* strains, PS/LS, and PS/LS-CL were mounted on conductive carbon tape and deposited a 10 nm gold–palladium alloy layer; the coated samples were examined via scanning electron microscopy (GeminiSEM 500, Carl Zeiss, Jena, Thüringen, Germany) operated at 10.00 kV accelerating voltage [30].

### 2.5. Encapsulation Efficiency

The encapsulation efficiency (EE) of the microcapsules for LJ, LA, and LGG was calculated based on the ratio of unencapsulated *Lactobacillus* to the total *Lactobacillus* content, as defined by the following equation:

(1) $$Encapsulation\;efficiency\;\left(\%\right)=\left(\frac{N_{0}-N}{N_{0}}\right)\times 100$$

where N_0_ is the total content of LJ, LA, and LGG before encapsulation, determined via plate counting (CFU/mL), and N (N_1_, N_2_) is the content of free cells (unencapsulated LJ, LA, and LGG) in the supernatant determined via plate counting (CFU/mL).

### 2.6. Survival Rate of Lactobacillus in PS/LS-CL and PS/LS After Simulated Digestion

To evaluate the gastrointestinal stability of the PS/LS-CL and PS/LS, 0.1 g of each sample was suspended in 0.9 mL of simulated gastric fluid (pH 2.0, containing 0.3% *w*/*v* pepsin) at a *w*/*v* ratio of samples to simulated gastric fluid of 1/9 and incubated at 37 °C for 2 h. After centrifugation at 6000× *g* for 10 min, the pellet was resuspended in 0.9 mL of simulated intestinal fluid (pH 6.8, containing 0.1% *w*/*v* pancreatin and 0.15% *w*/*v* bile salts) at a *w*/*v* ratio of samples to simulated intestinal fluid of 1/9 for a further 4 h incubation at 37 °C. Finally, 100 µL of the suspension was shaken by vortexing at high speed for 30 min, and then serially diluted and plated on MRS agar for viable colony counting after anaerobic incubation at 37 °C for 48 h. All samples were tested in triplicate under the same in vitro simulated digestion conditions.

### 2.7. Long-Term Storage

To assess long-term storage stability under commercial conditions, PS/LS and PS/LS-CL were sealed in tin foil bags using a vacuum sealing machine and then stored at 4 °C with 60% RH (relative humidity) for 90 days. Samples were aseptically collected at predetermined intervals (days 0, 15, 30, 60, and 90), subjected to serial dilution in sterile PBS (pH 7.4), and 100 µL of the suspension was shaken by vortexing at high speed for 30 min, serially diluted, and plated on MRS agar for viable colony counting after anaerobic incubation at 37 °C for 48 h.

### 2.8. In Vivo Colonization Efficacy of Novel Porous Starch/Lactobacillus Microcapsules

BALB/c mice (male, 6–8 weeks, n = 8/group; the pH of the mice stomach is approximately 2, primarily containing pepsin, while the pH of the mice intestine is around 7, mainly containing trypsin) were gavaged with 200 μL of phosphate-buffered saline (PBS) suspension (containing either 1 × 10^8^ CFU free *Lactobacillus* cells or an equivalent amount of CFU of PS/LS-CL) for 7 consecutive days. The control group received daily oral gavage of 200 μL phosphate-buffered saline (PBS, pH 7.4) for 7 consecutive days, maintaining equivalent handling procedures to experimental groups while excluding probiotic administration. Fresh fecal samples were collected 48 h after the completion of the gavage procedure, and then 0.1 g fecal sample was homogenized in 1 mL PBS by vortex mixing (2000 rpm, 30 min); 100 µL of the suspension was serially diluted and plated on MRS agar for viable colony counting after anaerobic incubation at 37 °C for 48 h. All the animal procedures were in accordance with the guidelines of the Care and Use of Laboratory Animals of Zhejiang University, and the experiments were approved by the ZJU-Laboratory Animal Welfare and Ethics Review Committee (No. ZJU20250251).

Total RNA was extracted from 0.1 g of fecal samples using RNAiso Plus following the manufacturer’s protocols. Quantitative real-time PCR (qRT-PCR) was conducted on the QuantStudio 7 Flex System (Applied Biosystems, Hayward, CA, USA) with SYBR Green Master Mix (Takara Bio, Kusatsu, Japan). The 16s universal primers and species-specific primers for the three *Lactobacillus* strains are listed in Table 1. The amplification conditions included the following: 95 °C for 30 s (initial denaturation), followed by 40 cycles of 95 °C for 5 s and 60 °C for 30 s. Melt curve analysis (60–95 °C, 0.3 °C/s) confirmed primer specificity. Relative mRNA expression was calculated using the 2^−ΔΔCT^ method with 16s as the endogenous control [31]. Gene expression fold changes between experimental (microencapsulated) and control (free *Lactobacillus*, and PBS) groups were statistically analyzed by Student’s *t*-test.

### 2.9. Statistical Analysis

All experimental determinations were carried out in triplicate. Statistical analyses were performed using SPSS (v22.0, IBM Corp., Armonk, NY, USA) and OriginPro (v9.1, OriginLab Corp., Northampton, MA, USA) software packages. Quantitative results are presented as mean values ± standard deviation (SD).

## 3. Results and Discussion

### 3.1. Morphology

**Figure 2:**
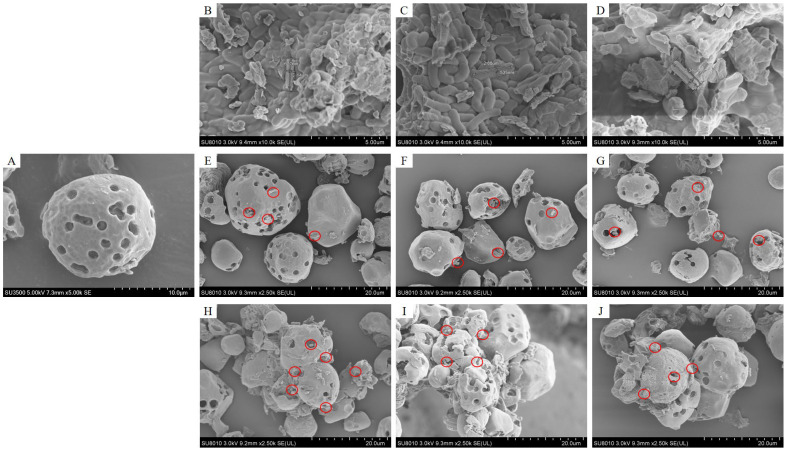
Electron micrographs of (**A**) porous starch (PS), (**B**) *Lactobacillus johnsonii* (LJ), (**C**) *Lactobacillus acidophilus* (LA), (**D**) *Lactobacillus rhamnosus GG* (LGG), (**E**) LJ encapsulated in PS (PS/LJ), (**F**) LA encapsulated in PS (PS/LA), (**G**) LGG encapsulated in PS (PS/LGG), (**H**) LJ encapsulated in PS via in situ cross-linking (PS/LJ-CL), (**I**) LA encapsulated in PS via in situ cross-linking (PS/LA-CL), (**J**) LGG encapsulated in PS via in situ cross-linking (PS/LGG-CL). The regions marked by red circles indicate *Lactobacillus* adhesion on the surface of porous starch or penetration into its micropores.

As demonstrated in Figure 2A–D, the porous starch (PS) prepared by the enzymatic hydrolysis method exhibited a uniform distribution of micropores, with pore sizes ranging from 1 to 3 μm. The dimensions of *Lactobacillus johnsonii* (LJ), *Lactobacillus acidophilus* (LA), and *Lactobacillus rhamnosus GG* (LGG) were measured as approximately 0.6 μm × 2.0 μm, 0.5 μm × 2.5 μm, and 0.7 μm × 2.3 μm, respectively. The cellular dimensions of the three *Lactobacillus* exhibited precise compatibility with the microporous architecture of porous starch (average pore diameter: 1–3 μm). This structural congruence enabled the efficient entrapment of probiotic cells within the porous starch microcavities. Figure 2E–G revealed that after porous starch encapsulation, a subset of LJ, LA, and LGG cells adhered to the starch surface, while some LJ, LA, and LGG were embedded within the pores. The physical adhesion of *Lactobacillus* to starch granules may be primarily mediated by surface-expressed adhesins, which act as molecular “anchors” to facilitate specific binding [11]. In addition, the rough and porous microstructure of starch granules offered numerous attachment sites for these adhesins. The novel porous starch/*Lactobacillus* microcapsules fabricated through in situ cross-linking exhibited pronounced aggregation phenomena (Figure 2H–J). This aggregation likely arose from intermolecular interactions mediated by (i) hydrogen bonding between starch hydroxyl groups and *Lactobacillus* surface glycans, and (ii) covalent cross-links formed via sodium trimetaphosphate (STMP)-activated phosphorylation of starch α-1,4/1,6 glycosidic bonds [11,24].

### 3.2. Encapsulation Efficiency

As shown in Table 2, the counts of LJ, LA, and LGG after porous starch encapsulation were (1.33 ± 0.16) × 10^9^, (5.18 ± 0.15) × 10^9^, and (1.73 ± 0.01) × 10^9^ CFU/mL, with encapsulation efficiencies of 67.92%, 58.68%, and 47.71%, respectively. These findings are consistent with the data reported by Xing et al. [20], demonstrating that porous starch serves as an effective encapsulation matrix for *Lactobacillus*. The novel PS/LJ microcapsules (PS/LJ-CL) fabricated via in situ cross-linking demonstrated a *Lactobacillus* count of (1.56 ± 0.08) × 10^9^ CFU/mL and an encapsulation efficiency of 79.56%, which markedly surpassed those of the PS/LJ. Similarly, PS/LA-CL and PS/LGG-CL demonstrated significant advantages over their conventional counterparts (PS/LA, PS/LGG), achieving a *Lactobacillus* count of (6.93 ± 0.20) × 10^9^ CFU/mL and (2.03 ± 0.02) × 10^9^ CFU/mL with encapsulation efficiencies of 78.49% and 55.96%, respectively. And the encapsulation efficiency of PS/LS-CL was significantly higher than that of *Lactobacillus* microcapsules prepared with sodium alginate (37.9%, 36.9%) [32] and those fabricated using phytic acid–chitosan complexes (41.1%) [33], which indicated that PS/LS-CL is an effective processing method for probiotic supplement foods.

This enhancement can be attributed to the cross-linking-mediated interparticle aggregation of porous starch facilitating the entrapment of surface-adhered *Lactobacillus* within the aggregated starch matrix, thereby significantly enhancing encapsulation efficiency through improved spatial confinement and reduced bacterial leakage. This is strongly corroborated by scanning electron microscopy observations, which visually confirmed the entrapment of *Lactobacillus* within the porous starch and the cross-linking-induced interparticle aggregation.

### 3.3. Storage Stability

**Figure 3:**
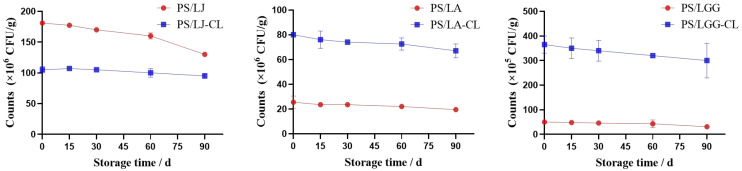
Survival *Lactobacillus* in PS/LS-CL and PS/LS during storage time (4 °C, 60% RH).

The storage stability of *Lactobacillus*-loaded microcapsules constitutes a critical quality attribute for probiotic supplements, as their therapeutic efficacy fundamentally depends on maintaining viable bacterial populations [34]. As shown in Figure 3, both the PS/LS and PS/LS-CL exhibited a progressive decline in viable counts of *Lactobacillus* strains with prolonged storage time, particularly after 60 days of storage. However, the PS/LS-CL group exhibited a slower decline rate in the viable bacterial survival rate compared to the PS/LS group, particularly in the PS/LJ-CL group. At the 90-day storage endpoint, the PS/LJ-CL achieved a viability retention of 90.48% versus the PS/LJ’s 71.82%, which aligned with its denser cross-linking network and superior protective efficacy. In addition, compared to *Lactobacillus bulgaricus* microcapsules encapsulated with whey protein [35], LA microcapsules co-coated with gelatin and gum Arabic [36], and LGG microcapsules co-encapsulated with alginate and pectin, the PS/LS-CL demonstrates enhanced long-term storage stability [37]. Collectively, PS/LS-CL exhibited moderate efficacy in enhancing *Lactobacillus* storage stability compared to the PS/LS, although, for the LA and LGG strains, no statistically significant advantage was observed between the in situ cross-linking PS/LS-CL groups and the PS/LS groups.

### 3.4. Survival of Encapsulated Lactobacillus in Simulated Digestion

The ability of *Lactobacillus*-encapsulated microcapsules to withstand gastrointestinal challenges (e.g., gastric acid at pH 2.0 and intestinal fluid containing 0.3% bile salts) is a critical determinant for their targeted delivery to the colon and subsequent colonization efficacy, as this survivability directly governs the proportion of viable bacteria reaching the intestinal niche [38,39]. As shown in Table 3, after sequential exposure to simulated gastric fluid (SGF, pH 2.0) for 2 h and simulated intestinal fluid (SIF, 0.3% bile salts) for 4 h, the PS/LJ exhibited a viability of 3.04 ± 0.15%, whereas PS/LA and PS/LGG demonstrated significantly lower survival rates (<0.1%). This disparity may stem from the intrinsic tolerance of LJ strains to gastric acid and bile salts [40]. Niu et al. also demonstrated that *Lactobacillus johnsonii* exhibited significantly higher acid tolerance than the other three *Lactobacillus* strains, which may be because *Lactobacillus johnsonii* possessed a unique repertoire of genes specifically associated with acid tolerance [41,42]. The viability rates of PS/LJ-CL, PS/LA-CL, and PS/LGG-CL were 12.19%, 5.63%, and 4.8%, respectively, indicating the enhanced protective capacity of the cross-linked porous starch/*Lactobacillus* microcapsules. This phenomenon may be attributed to the interparticle aggregation-induced pore shielding effect within cross-linked starch matrices [27,43], which partially occludes micropores and creates a denser network and protective niches for bacterial survival. Li et al. also found that the cross-linking modification of starch significantly enhanced the structural integrity and digestive resistance of microcapsules [43]. Moreover, the survival rate of PS/LS-CL microcapsules after simulated digestion was higher than that of LA and LGG microcapsules prepared with alginate [44], *Lactobacillus plantarum* microcapsules based on cellulose polymers [45], and LGG microcapsules co-encapsulated with alginate and pectin [37]. High tolerance to in vitro simulated digestion demonstrates that this technology offers potential improvements for excipient formulations and processing protocols in probiotic food products.

### 3.5. In Vivo Colonization Efficacy

**Figure 4:**
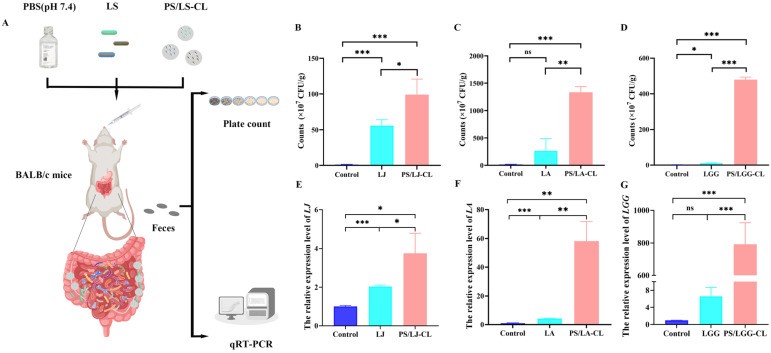
In vivo colonization detection in mouse colon after 7-day oral gavage. (**A**) BALB/c mice (6–8 weeks old) received daily oral gavage for 7 consecutive days, and their fresh fecal samples were aseptically collected 48 h after the final administration, immediately snap-frozen in liquid nitrogen, and stored at −80 °C until analysis. The control group: daily oral gavage of 200 μL 0.01 M phosphate-buffered saline (PBS, pH 7.4); LS groups (LJ, LA, LGG) daily oral gavage of 200 μL PBS containing free *Lactobacillus* cells (1 × 10^8^ CFU); PS/LS-CL groups (PS/LJ-CL, PS/LA-CL, PS/LGG-CL): daily oral gavage of 200 μL PBS containing *Lactobacillus* cells (1 × 10^8^ CFU). (**B**–**D**) the abundance of LJ, LA, and LGG in fecal samples via gradient dilution plate counting. (**E**–**G**) the abundance of LJ, LA, and LGG in fecal samples via qRT-PCR. ns: not significant; * *p* < 0.05, ** *p* < 0.01, *** *p* < 0.001.

The colonization capacity of encapsulated *Lactobacillus* strains serves as a pivotal determinant for successful probiotic delivery, given that their physiological niche predominantly resides in the colonic region [46]. To further investigate the colonization efficacy of novel microcapsules, specific pathogen-free (SPF) mice were orally administered the PS/LS-CL via gastric gavage for 7 consecutive days, as described in Figure 4A.

As depicted in Figure 4, both the free *Lactobacillus* gavage group and the PS/LS-CL gavage group demonstrated significantly enhanced colonization efficiency compared to the control group (*p* < 0.05). The colony enumeration data presented in Figure 4B–D revealed that the amount of LJ was 5.5 × 10^7^ CFU/g in the free LJ gavage group, which increased to 9.9 × 10^7^ CFU/g in the PS/LJ-CL gavage group. Similarly, the amount of LA increased from 2.6 × 10^9^ CFU/g in the free LA gavage group to 1.3 × 10^10^ CFU/g in the PS/LA-CL group. For LGG, the amount rose from 1.0 × 10^8^ CFU/g to 2.8 × 10^9^ CFU/g in the PS/LGG-CL group compared to the free LGG gavage group. The results of quantitative real-time PCR analysis of fecal samples described in Figure 4E–G also showed that the abundance of LJ in the PS/LJ-CL group was approximately 2-fold higher than that in the free LJ group (*p* < 0.05), the relative abundance of LA in the PS/LA-CL group was more than 10-fold higher than that in the free LA group (*p* < 0.01), and the expression level of LGG specific genes in PS/LGG-CL was nearly 80-fold higher than that in the free LGG group (*p*< 0.001).

The higher abundance of *Lactobacillus* in the PS/LS-CL group indicated that the novel porous starch/*Lactobacillus* microcapsules effectively shielded *Lactobacillus* from gastric and enteral degradation and achieved microbial colonization, which was consistent with the in vitro simulated digestion results. Numerous studies have demonstrated that resistant starch (RS), as a dietary fiber, undergoes microbial degradation in the colon through enzymatic fermentation by gut microbiota [47], and the degraded starch and cross-linked starch derivatives (e.g., oligosaccharides, short-chain fatty acids) generated during microcapsule disintegration function as bioavailable carbon substrates, fueling glycolytic metabolism in *Lactobacillus* and enhancing their proliferation [48,49].

Notably, the colonization-enhancing efficacy of the PS/LJ-CL group for *Lactobacillus* was comparatively moderate when contrasted with the PS/LA-CL and PS/LGG-CL groups, which may be because the effective supplementation and colonization of *Lactobacillus* are also associated with the ability of the intrinsic gastric acid and bile salt resistance of *Lactobacillus*. Debbie et al. also found that enhancing bile acid tolerance through genetic engineering significantly improved the survival rate and gastrointestinal persistence of *Bifidobacterium* and *Lactococcus* strains in murine models [50]. These findings highlight that the novel porous starch-based encapsulation enhances the colonization capacity of acid- and bile salt-sensitive *Lactobacillus* strains, providing a viable solution for formulating food supplements with such sensitive probiotics.

## 4. Conclusions

In this study, we successfully developed new porous starch/*Lactobacillus* microcapsules (PS/LS-CL: PS/LJ-CL, PS/LA-CL, PS/LGG-CL) via in situ cross-linking. The cross-linking of *Lactobacillus*-encapsulated porous starch formed covalent starch–starch and starch–*Lactobacillus* networks, leading to structural optimization and significantly enhancing encapsulation efficiency. The new porous starch encapsulation strategy not only maintained a viable survival rate exceeding 80% for *Lactobacillus* under prolonged storage but also conferred enhanced protection against harsh gastrointestinal conditions (e.g., low pH and bile salt stress). In vivo studies demonstrated that the PS/LS-CL gavage group exhibited the superior colonic colonization efficacy of *Lactobacillus* compared to the free *Lactobacillus* gavage group, particularly for acid- and bile salt-sensitive strains. Although this study demonstrated that PS/LS-CL exhibited significant advantages in the delivery of *Lactobacillus* in both in vitro and in vivo mouse experiments, in vivo experiments using rat models and large animals such as beagle dogs are necessary to further validate the effectiveness. Overall, our method obviates the necessity of intricate post-processing procedures, thereby expediting the translation of research outcomes into actionable deliverables. Moreover, it offers novel perspectives on the intestinal delivery of probiotics and provides innovative strategies and a theoretical basis for the development of future probiotic supplement products in food products.

## Figures and Tables

**Table 1:** 16s universal primers and specific primers for three *Lactobacillus*.

Samples	Forward 5′-3′	Reverse 5′-3′
Universal Eubacteria 16s	CGGCAACGAGCGCAACCC	CCATTGTAGCACGTGTGTAGCC
LJ	TCGAGCGAGCTTGCCTAGATGA	TCCGGACAACGCTTGCCACC
LA	AAGAGGCTAAGGCTAAGGG	TGAATAACGAAGTCACCACC
LGG	CACCGATTGTTCCAGCAGTTTAT	GCTTCATCAGTCAGCCTTCCTTTT

**Table 2:** Encapsulation yield and efficiency of three *Lactobacillus*.

Samples	The Total Amount of *Lactobacillus* (N_0_) (CFU/mL)	Free Cells (N; N_1_, N_2_) (CFU/mL)	The Amount of Embedded *Lactobacillus* (CFU/mL)	Encapsulation Efficiency (%)
PS/LJ	1.96 ± 0.17 × 10^9^	0.63 ± 0.01 × 10^9^ a	1.33 ± 0.16 × 10^9^ a	67.92 ± 8.16
PS/LJ-CL	1.96 ± 0.17 × 10^9^	0.40 ± 0.09 × 10^9^ b	1.56 ± 0.08 × 10^9^ a	79.56 ± 4.08
PS/LA	8.83 ± 0.13 × 10^9^	3.65 ± 0.03 × 10^9^ a	5.18 ± 0.15 × 10^9^ b	58.68 ± 1.70
PS/LA-CL	8.83 ± 0.13 × 10^9^	1.90 ± 0.06 × 10^9^ b	6.93 ± 0.20 × 10^9^ a	78.49 ± 2.27
PS/LGG	3.63 ± 0.07 × 10^9^	1.90 ± 0.06 × 10^9^ a	1.73 ± 0.01 × 10^9^ b	47.71 ± 0.28
PS/LGG-CL	3.63 ± 0.07 × 10^9^	1.60 ± 0.05 × 10^9^ b	2.03 ± 0.02 × 10^9^ a	55.96 ± 0.55

Where different lowercase letters (a, b) denote statistically significant differences (*p* < 0.05) between the PS/LS-CL and PS/LS, as determined by Duncan’s multiple range test.

**Table 3:** Survival rate of *Lactobacillus* in PS/LS-CL and PS/LS after in vitro simulated gastrointestinal digestion (simulated gastric fluid (pH 2.0, containing 0.3% *w*/*v* pepsin) incubated at 37 °C for 2 h; simulated intestinal fluid (pH 6.8, containing 0.1% *w*/*v* pancreatin and 0.15% *w*/*v* bile salts) incubated at 37 °C for 4 h).

Samples	Initial Count (CFU/g)	The Count of Bacteria After Simulated Digestion (CFU/g)	Survival Rate (%)
PS/LJ	1.80 ± 0.23 × 10^8^	5.47 ± 0.17 × 10^6^ b	3.04
PS/LJ-CL	1.07 ± 0.06 × 10^8^	1.30 ± 0.07 × 10^7^ a	12.19
PS/LA	2.55 ± 0.35 × 10^8^	<1 × 10^5^ b	<0.1
PS/LA-CL	8.00 ± 0.10 × 10^8^	4.50 ± 0.03 × 10^7^ a	5.63
PS/LGG	5.00 ± 0.20 × 10^6^	<1 × 10^4^ b	<0.1
PS/LGG-CL	3.65 ± 0.25 × 10^7^	1.75 ± 0.25 × 10^6^ a	4.8

Where different lowercase letters (a, b) denote statistically significant differences (*p* < 0.05) between the PS/LS-CL and PS/LS, as determined by Duncan’s multiple range test.

## Data Availability

The original contributions presented in the study are included in the article, further inquiries can be directed to the corresponding author.
